# Nutritional Quality of Plant-Based Meat and Dairy Imitation Products and Comparison with Animal-Based Counterparts

**DOI:** 10.3390/nu15020401

**Published:** 2023-01-12

**Authors:** Alexandra Katidi, Konstantina Xypolitaki, Antonis Vlassopoulos, Maria Kapsokefalou

**Affiliations:** Laboratory of Chemistry and Food Analysis, Department of Food Science and Human Nutrition, Agricultural University of Athens, 11855 Athens, Greece

**Keywords:** alternative protein sources, meat imitations, meat substitutes, dairy imitations, dairy substitutes, plant-based diet, vegan, branded food composition database, nutritional composition, Nutri-Score

## Abstract

While consumers are increasingly adopting plant-based meat and dairy imitation products, the nutritional quality and adequacy of those foods to act as a substitute is still under discussion. The Greek Branded Food Composition Database (HelTH) was expanded to map currently available meat and dairy imitations in Greece. Their main ingredient used, nutritional composition, and promotion as a healthy, nutritious food were described, and their overall nutritional quality using the Nutri-Score algorithm was evaluated. A total of *n* = 421 plant-based imitations were analyzed, made primarily of wheat or wheat mixes (83.5%) for meat imitations and grain (19.8%) or vegetable oil (17.1%) for dairy imitations. All meat imitations were high in protein and fiber, while, for dairy, only yogurts carried a protein content claim (80.9%). Imitation sausages, milk, and yogurt products had lower total fat and saturated fat content compared to their animal-based counterparts. All dairy imitations had lower protein content than animal-based dairy. The nutritional quality of imitation cheeses was graded as D–E, under the Nutri-Score system, compared to A–C for the animal-based cheese. Plant-based imitations have variable composition based on their main ingredient, and the substitution of specific food groups with plant-based alternatives may not support an equivalent or improved diet.

## 1. Introduction

Non-communicable diseases, food insecurity, and climate change are major worldwide concerns related, at least partially, to dietary choices. Therefore, sustainable dietary practices are advocated by scientists, policymakers, and food developers [1,2]. In particular, the transition from animal-based to plant-based diets, a term that describes dietary patterns that emphasize foods derived from plant sources coupled with lower consumption or exclusion of animal products [3,4,5,6], is driven by the simultaneous crises relating to public health, environmental pressures, as well as other social and ethical issues including animal welfare [7,8,9,10,11,12].

Scientific consensus as well as consumer interest in plant-based diets has prompted food sector innovations [3,13]. Meat and dairy imitation products that capitalize on this trend have been developed [13]. These are based on the so-called alternative proteins, namely proteins derived from plants (for example, grains, legumes, and nuts), fungus (mushrooms), algae, insects, and even cultured (lab-grown) meat [13,14,15,16,17,18,19]. Meat and dairy imitations have physical and sensory characteristics (taste, texture, visual appearance, and cooking method) that resemble those of their animal-based counterparts [3] but may lack nutritional quality. These products are often poorer in protein and rich in calories, salt, fat, fiber, and total carbohydrates; nevertheless, their overall evaluation is limited [3,13,14,15,17,20,21,22,23]. There is a knowledge gap of fundamental significance to multiple stakeholders on whether meat and dairy imitations resemble their animal-based counterparts regarding nutritional aspects and whether the alternative protein source that their matrix is based on is important in determining the nutritional profile of the products.

Consumer acceptance of meat and dairy imitations is related to several factors, including cost, convenience, familiarity, and cultural and demographic factors [16,24]; however, overall, meat and dairy imitations are gaining popularity, particularly the plant-based products. Thus, their contribution to dietary intake is becoming significant. This suggests that robust research efforts must address questions related to their nutritional quality and profile in order to investigate potential consequences of the consumption of meat and dairy imitations for public health.

In line with scientific consensus and consumer interest in plant-based diets [13], the foodscape is now populated with plant-based meat and dairy imitation products; however, studies that systematically analyze their nutritional composition have not been performed. Thus, our study presented herein aims to contribute new knowledge and to evaluate the nutritional quality and the main ingredients used in plant-based meat and dairy imitations sold in Greece.

The objectives of the study are (1) to describe meat and dairy imitation products in terms of their content, nutrition claims, quality indicators, and nutritional composition collectively and according to their main alternative protein source and (2) to compare their nutritional profile with their animal-based counterparts.

## 2. Materials and Methods

### 2.1. Data Collection and Data Sources

The Hellenic Food Thesaurus (HelTH), the Greek branded food composition database (BFCD), was the main data source used to identify plant-based imitation products and their animal-based counterparts [25,26,27,28]. Briefly, HelTH collects and curates food data as presented on-pack through online sampling of foods available in main online supermarkets in Greece [29]. HelTH was launched in 2019 by the Agricultural University of Athens. It included, for *n*= 4002 food items, data on the nutritional composition of foods, any health and/or nutrition claims, other quality indicators presented on-pack (bio/organic, environmental claims, etc.), graphical indicators (logos), and allergens. In June 2022, HelTH contained data for *n* = 49 plant-based meat and dairy imitations. A targeted expansion of HelTH to increase the number of plant-based meat and dairy imitations was carried out from June to October 2022 via thirteen online supermarkets following the same sampling methodology as the 2019 version of HelTH [29]. Two of these supermarket chains represent more than 50% of the total Greek market share, while six of the selected retailers are targeted to vegan and bio/organic products. The sum of the thirteen specific retailers was chosen in an effort to reflect choices available to the majority of Greek consumers.

Products eligible for the expansion included those sold as meat and dairy imitations. In detail, the following food products were considered eligible under the main meat and dairy imitation categories (Table 1). An internet search was conducted through the selected supermarket websites using keywords such as “meat alternatives”, “meat substitutes”, “meat-free”, “dairy alternatives”, “dairy substitutes”, “dairy-free”, “plant-based”, “vegan”, and “vegetarian” to ensure all available products were captured.

### 2.2. Nutritional Composition and Profiles

The nutritional profiles of all products were analyzed in terms of their nutritional composition in key macronutrients, the presence of nutrition claims, and other quality indicators on-pack as well as their performance under the Nutri-Score nutrient profiling scheme.

For each product, data on the nutritional declaration per 100 g or 100 mL, ingredients, health and nutrition claims, and any additional logos and endorsements were transcribed from photographs. All data were checked for any inconsistencies and errors by a second, independent reviewer.

The Nutri-Score algorithm was calculated for each food based on its nutritional composition per 100 g of food, as previously described [30]. In brief, under the Nutri-Score algorithm, the content of energy (kJ), total sugars (g), saturated fatty acids (SFAs) (g), and sodium (mg) (negative nutrients) is scored from 0 to 10 with higher scores for higher content, and the content of protein (g), fiber (g), and fruits/vegetables/pulses/nuts/specific oils (FV%) (positive nutrients) is scored from 0 to 5 with higher scores for higher content. The Individual Nutrient scores are combined to derive the overall FSAm-NPS score which ranges from −15 to +40 and is calculated by the subtraction of positive scores from the negative nutrients’ scores. For the calculation of the FSAm-NPS score, fiber and FV% scores are considered eligible for all foods, but the protein score is only included in the calculation if the sum of negative nutrient scores is <11, if a food has a FV% Score = 5, and for the calculation of the FSAm-NPS score of cheeses [31]. The FSAm-NPS score is translated into Nutri-Score grades based on the following criteria for solids: (A) was assigned to foods with a score from −15 to −1, (B) to foods with a score from 0 to 2, (C) to foods with a score 3 to 10, (D) to foods from 11 to 18, and (E) to foods from 19 to 40 [30]. FV% was estimated based on the ingredient list in a two-step process. Firstly, all foods were screened to assess the presence of at least 40% content in fruits, vegetables, pulses, nuts, rapeseed, walnut, and olive oils, which is the minimum content required. Then, for the products that met this minimum requirement, a thorough quantification was carried out. For the purpose of this study, specifications of the Nutri-Score algorithm were considered [31]. Plant-based imitations contain vegetables, nuts, fruits, pulses, and oils in their ingredient list. However, before awarding positive points for the FV% of the food, the processing method and the form of the final product of fruits/vegetables/pulses/nuts/specific oils should be considered. For example, specific criteria have been set for coconut, which was counted as fruit only in the form of fresh coconut product. Furthermore, concentrated fruit juices, powders, candied fruits, and flours were not considered as fruits/vegetables/pulses/nuts. Plant-based beverages were regarded as solid foods at the calculation of the Nutri-Score algorithm, as indicated in the Nutri-Score algorithm [31]. Products that did not contain any data about energy, SFA, total sugar, or sodium were excluded, as no Nutri-Score could be calculated. Missing nutrient values could be due to lack of nutritional declaration or low-quality images obtained from the specific foods. On the contrary, for “positive nutrients”, missing information was imputed as zero.

### 2.3. Statistical Analysis

Statistical analysis was carried out using IBM SPSS Statistics^®^ (version 23, Northridge, CA, USA). Nutritional composition data (content per 100 g or 100 mL of product) and the FSAm-NPS score were analyzed as continuous variables. Data were tested for normality using the Kolmogorov–Smirnov test. None of the variables followed the normal distribution. Therefore, variables were expressed as median (interquartile range). Differences were tested using the Kruskal–Wallis non-parametric test for k independent samples. Between-group differences were tested using the Mann–Whitney U test for continuous variables. Statistical significance was set at 0.01% to adjust for multiple comparisons (Bonferroni correction).

## 3. Results

A total of 421 meat and dairy imitation products were collected from the online retailers. Two products were excluded from the analysis as they were plant-based products but they were not marketed as meat imitations (e.g., falafels). Meat and dairy imitations were grouped under their respective animal-based categories, according to their use and positioning (e.g., milk, yogurt, chicken imitation, etc.) (Table 1). All imitation products were plant-based; there were no products based on other novel protein sources such as algae or insects. However, there were multiple protein sources identified, within the plant-based range, and the main ingredient used differed per category and sub-category.

### 3.1. Description of Meat and Dairy Imitation Products

**Figure 1:**
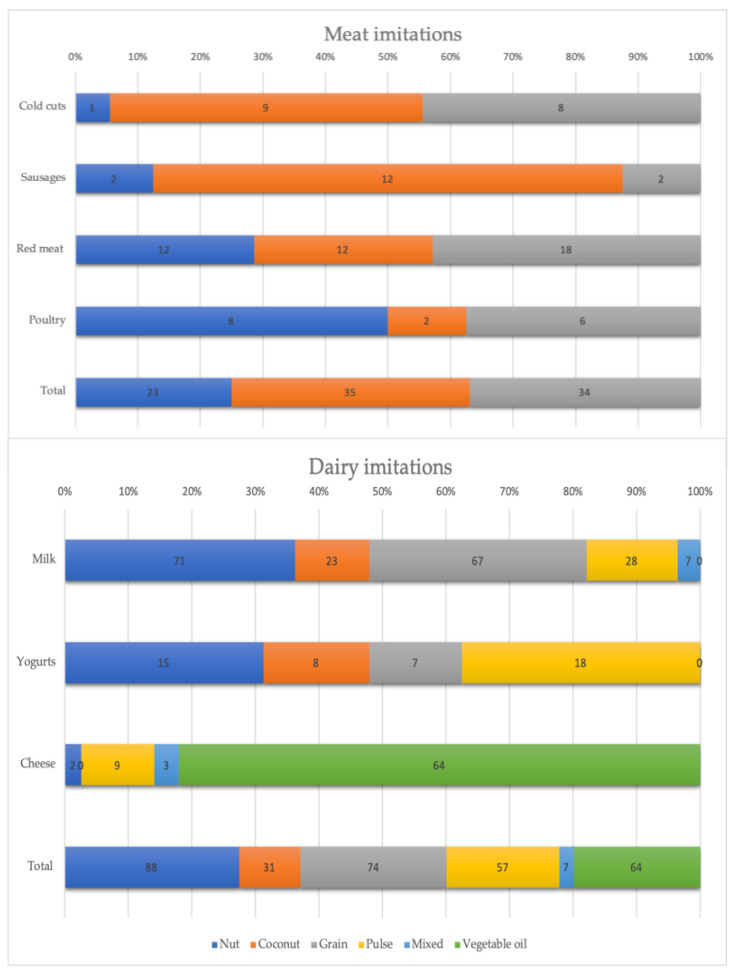
Percentage of products that include protein-containing ingredients for imitation meat and dairy categories.

Meat imitation products were based on wheat/seitan (38.0%), other pulses/mix (36.9%), or soy/tofu (25.0%) (Figure 1). Most plant-based cold cuts and sausage imitations were based on wheat/seitan (50% and 75%, respectively), most poultry imitations were based on soy/tofu (50%), while the majority of plant-based red meat imitations was based on a mixed matrix (42.9%). Dairy imitation products were based on nuts (36.2%), coconut (11.7%), grain (34.1%), pulse (14.2%), vegetable oil (20.5%), or a mixed matrix (2.7%). In particular, milk imitation products were based on nuts (36.2%), followed by those based on grains (34.1%), coconut (11.7%), pulses (14.2%), and another or a mixed matrix (3.6%). Yogurt imitation products were based on pulses (37.5%), nuts (31.3%), coconut (16.7%), and grains (14.6%). Cheese imitation products were mainly based on vegetable oils (83.1%) (Figure 1).

### 3.2. Claims and Quality Indicators on Plant-Based Imitations

The majority of meat and dairy imitations featured extensive on-pack information that included nutrition claims (65%) and other claims—vegan diets (62.3%), allergen-free (39.6%), naturalness (25.2%), and bio/organic (37.3%). None of the imitation products under investigation made any sort of health claim (Table 2).

Nutrition claims most often utilized for meat imitations were those for protein (41.7%), fiber (18.8%), and vitamins and minerals (9.4%). Poultry imitations was the category with the highest frequency of protein claims (68.8%), fiber claims (37.5%), and vitamin and minerals claims (25%) (Table 2). Cold cuts and sausages imitations bore a bio/organic label on their packaging (83.3% and 68.4%, respectively). Natural claims were present in all meat imitations categories, with the highest prevalence found in the red meat imitations (27.9%). Allergens-free claims were also detected in all meat imitations categories. Gluten-free claims were present for 23.3% of red meat imitations, 21.1% of sausage imitations, 16.7% of cold cuts imitations, and 12.5% of poultry imitations. Soy-free claims were present for 11.1% of cold cuts imitations, 18.6% of red meat imitations, 12.5% of poultry imitations, and 5.3% of sausage imitations. A vegan/vegetarian claim was present for 82.3% of the meat imitations, while 60.4% of these products carried a meat-free claim (Table 2).

In dairy imitation products, 19.1% of yogurt imitations and 10.4% of milk imitations carried a protein claim. In milk imitations, 58.1% carried a sugar claim, 15.4% carried a fat claim, 10.8% a fiber claim, 17.4% a vitamin claim, and 20.7% a minerals claim. Thirty-seven percent of dairy imitations were bio/organic, and 26.5% bore a natural claim. Forty-five percent of plant-based yogurts were gluten-free, while all dairy imitations categories carried a soy-free claim, in different percentages. The prevalence of dairy imitations that claimed to be vegan/vegetarian was 57.1%, while 38.6% claimed to be dairy-free (Table 2).

**Table 2:** Prevalence of meat and dairy imitation products bearing nutrition claims and other quality indicators on their packaging.

Imitation Products	Protein Claim *n* (%)	Sugar Claim *n* (%)	Fat Claim *n* (%)	Fiber Claim *n* (%)	Vitamin Claim *n* (%)	Minerals Claim *n* (%)	Vegan/Vegetarian *n* (%)	Meat-Free *n* (%)	Dairy- Free *n* (%)	Gluten-Free *n* (%)	Soy-Free *n* (%)	Bio/Organic *n* (%)	Natural *n* (%)
Cold Cuts (*n* = 18)	5 (27.8)	-	-	2 (11.1)	-	-	16 (88.9)	12 (66.7)	7 (38.9)	3 (16.7)	2(11.1)	15 (83.3)	2 (11.1)
Sausages (*n* = 21)	8 (42.1)	-	-	2 (10.5)	-	-	18 (94.7)	15 (78.9)	8 (42.1)	4 (21.1)	1 (5.3)	13 (68.4)	2 (10.5)
Red meat (*n* = 43)	16 (37.2)	-	4 (9.3)	8 (18.6)	5 (11.6)	5 (11.6)	31 (72.1)	21 (48.8)	5 (11.6)	10 (23.3)	8 (18.6)	10 (23.3)	12 (27.9)
Poultry (*n* = 16)	11 (68.8)	-	2 (12.5)	6 (37.5)	4 (25)	4 (25)	14 (87.5)	10 (62.5)	2 (12.5)	2 (12.5)	2 (12.5)	-	3 (18.8)
Milk (*n* = 233)	25 (10.4)	140 (58.1)	37 (15.4)	26 (10.8))	42 (17.4)	50 (20.7)	130 (53.9)		72 (29.9)	691 (28.60.5)	72 (2.90.9)	111 (46.1)	64 (26.6)
Yogurts (*n* = 55)	9 (19.1)	10 (21.3)	5 (10.6)	-	7 (14.9)	7 (14.9)	23 (48.9)	23 (48.9)	10 (21.3)	21 (44.7)	13 (27.7)	12 (25.5)	11 (23.4)
Cheese (*n* = 85)	-	-	-	-	-	-	60 (70.6)	60 (70.6)	62 (72.9)	-	46 (54.1)	14 (16.5)	24 (28.2)

Percentages (%) are obtained per table raw by dividing the number of products that bear a claim/quality indicator with the number of products included in each imitation products category.

### 3.3. Nutritional Composition of Meat and Dairy Imitation Products

The nutritional composition of meat and dairy products and their imitations is presented in Table 3 and Table 4.

In imitation products, regarding the energy content, sausage imitations presented the highest median (247 kcal/100 g), followed by red meat imitations (231 kcal/100 g), cold cuts imitations (222 kcal/100 g), and poultry imitation products (220 kcal/100 g). All meat imitations were high in protein and high in fiber, too. Sausage and poultry imitations were both low in SFA, while plant-based cold cuts were high in salt (Table 3). Related to plant-based dairy imitations, plant-based beverages were low in SFA, and yogurt imitations had a low total and saturated fat content. In contrast, plant-based cheese imitations were high in total fat, SFA, and salt (Table 4). We also compared the nutritional composition of plant-based meat and dairy imitations with their animal-based counterparts, per meat and dairy category, in energy and key nutrients (protein, total fat, SFA, carbohydrates, sugars, fiber, salt). Plant-based cold cuts had a higher content of sugars compared to their animal-based counterparts (*p* = 0.001). Plant-based red meat imitations did not differ from their animal-based counterparts in energy or any key nutrient. Related to poultry meat, imitation products were higher in carbohydrates from their animal-based counterparts (*p* = 0.005). Sausages were the meat category that presented the most differences between plant-based and animal-based products. Particularly, sausage imitations were higher in protein and lower in salt, total fat, and saturated fat, compared to their animal-based equivalents.

In dairy products, differences were observed in all categories. Particularly, plant-based milk imitations were lower in energy, fat, SFA, and sugars but also had a lower protein content. Plant-based yogurt imitations were lower in SFA and higher in fiber but were also higher in carbohydrates and sugars and lower in protein, compared to their animal-based equivalents. Plant-based cheese imitations had a higher content of SFA, carbohydrates, and fiber as well, and they were also lower in protein, compared to animal-based cheese products.

**Table 3:** Nutritional composition of meat and meat imitation categories.

Meat Categories	Cold Cuts			Sausages			Red Meat			Poultry		
Nutrients	Plant-Based (*n* = 18)	Animal-Based (*n* = 66)	*p*-Value	Plant-Based (*n* = 19)	Animal-Based (*n* = 27)	*p*-Value	Plant-Based (*n* = 43)	Animal-Based (*n* = 6)	*p*-Value	Plant-Based (*n* = 16)	Animal-Based (*n* = 14)	*p*-Value
Energy (kcal)	221.5 (180.0, 248.3)	169.5 (101.5, 273.0)	0.194	247.0 (211.0, 269.0)	247.0 (224.0, 300.5)	0.2885	231.0 (195.0, 252.0)	213.0 (183.3, 234.3)	0.522	219.5 (190.3, 247.3)	194.0 (189.5, 230.5)	0.393
Protein (g)	27.3 (5.2, 32.2)	14.5 (12.7, 21.8)	0.055	25.2 (19.4, 30.6)	13.5 (12.2, 15.0)	<0.001	17.6 (14.9, 24.0)	12.9 (11.3, 18.5)	0.041	12.2 (9.4, 14.0)	16.4 (12.8, 18.4)	0.019
Fat (g)	12.2 (8.4, 16.0)	10.0 (2.3, 22.0)	0.322	12.5 (10.3, 15.0)	20.1 (15.5, 25.5)	<0.001	12.5 (7.6, 15.2)	15.6 (10.6, 17.0)	0.206	8.0 (7.3, 12.0)	9.6 (9.0, 11.9)	0.289
SFA (g)	1.8 (1.2, 3.6)	3.0 (0.8, 7.0)	0.479	1.3 (1.0, 7.4)	7.5 (5.5, 9.8)	<0.001	1.8 (0.9, 7.9)	6.7 (4.2, 8.2)	0.215	1.0 (0.7, 3.0)	3.7 (3.0, 4.2)	0.016
Carbo-hydrates (g)	5.4 (2.9, 6.5)	4.0 (1.2, 6.0)	0.134	4.2 (3.6, 5.6)	2.8 (1.0, 5.9)	0.04	7.0 (4.1, 10.5)	6.7 (2.0, 8.1)	0.488	15.9 (13.3, 20.0)	11.0 (58.0, 13.5)	0.005
Sugars (g)	1.7 (0.8, 2.4)	0.9 (0.0, 1.1)	0.001	0.6 (0.5, 2.0)	0.9 (0.48, 1.2)	0.956	1.1 (0.6, 2.0)	1.1 (0.8, -)	0.658	1.4 (0.6, 2.6)	0.6 (0.5, 1.2)	0.086
Fiber (g)	4.4 (4.1, 5.0)	0.0 (0.0, 0,0)	0.043	3.2 (0.7, 4.5)	1.5 (1.3, -)	0.456	4.1 (2.0, 5.7)	-	-	4.7 (2.3, 6.1)	3.7 (0.4, -)	0.354
Salt (g)	2.1 (1.8, 2.6)	2.5 (2.2, 2.8)	0.026	1.5 (1.3, 1.8)	2.3 (1.8, 2.5)	<0.001	1.3 (1.0, 1.8)	1.2 (1.2, 1.6)	0.811	1.3 (1.0, 1.6)	1.5 (1.3, 1.7)	0.271

Values shown are median (Q1, Q3).

**Table 4:** Nutritional composition of dairy and dairy imitation categories.

Dairy Categories	Milk			Yogurt			Cheese		
Nutrients	Plant-Based (*n* = 221)	Animal-Based (*n* = 119)	*p*-Value	Plant-Based (*n* = 40)	Animal-Based (*n* = 137)	*p*-Value	Plant-Based (*n* = 80)	Animal-Based (*n* = 172)	*p*-Value
Energy (kcal)	46.0 (32.8, 57.0)	63.0 (46.0, 65.0)	<0.001	80.0 (69.8, 97.5)	78.0 (69.0, 97.0)	0.034	283.0 (248.0, 305.0)	302.5 (247.0, 361.0)	0.034
Protein (g)	0.7 (0.5, 1.2)	3.3 (3.3, 3.5)	<0.001	2.1 (1.0, 3.8)	6.2 (4.8, 8.2)	<0.001	0.5 (0.0, 1.6)	23.0 (16.0, 26.0)	<0.001
Fat (g)	1.6 (1.2, 2.2)	1.6 (1.5, 3.5)	0.001	2.3 (1.9, 4.7)	2.0 (1.6, 4.4)	0.028	23.0 (20.0, 24.0)	24.0 (17.6, 29.0)	0.028
SFA (g)	0.2 (0.2, 0.4)	1.1 (0.8, 2.2)	<0.001	0.4 (0.3, 0.7)	1.3 (1.0, 2.7)	0.001	20.5 (15.8, 21.0)	16.0 (11.5, 20.0)	0.001
Carbs (g)	5.7 (2.5, 9.0)	4.7 (4.7, 5.1)	0.362	11.8 (5.6, 15.0)	5.2 (4.0, 8.7)	<0.001	21.0 (11.5, 23.0)	0.5 (0.0, 1.9)	<0.001
Sugars (g)	3.4 (1.3, 6.0)	4.7 (4.6, 5.1)	<0.001	8.5 (0.8, 11.0)	5.1 (4.0, 8.7)	<0.001	0.0 (0.0, 0.5)	0.3 (0.0, 1.0)	<0.001
Fiber (g)	0.6 (0.4, 1.1)	0.4 (0.0, -)	0.055	1.0 (0.5, 1.4)	0.0 (0.0, 0.8)	<0.001	1.9 (0.5, 2.9)	0.0 (0.0, 0.0)	<0.001
Salt (g)	0.1 (0.1, 0.1)	0.1 (0.1, 0.1)	0.301	0.1 (0.1, 0.1)	0.1 (0.1, 0.2)	0.359	1.9 (1.7, 2.1)	1.8 (1.4, 2.2)	0.359

Values shown are median (Q1, Q3).

In addition, we compared the nutritional composition of meat imitations and dairy imitations, according to their main ingredient (Table 5 and Table 6). For meat imitations, no differences were observed between soy-, wheat-, or mixed-based cold cuts or red meat or poultry imitations. Sausage imitations though, presented differences in their protein content. Particularly, wheat-based sausage imitations were higher in protein than soy-based and mixed sausage imitations. In contrast, multiple differences were observed between plant-based dairy imitations’ categories, depending on their matrix. Plant-based milk imitations differed from each other in energy, protein, total fat, SFA, carbohydrates, sugars, and fiber. Coconut milk presented the lowest content in energy, while pulse-based milk imitations were the highest in protein. Differences among plant-based yogurt imitations could be found in the protein, SFA, and salt content. In particular, protein content was the highest in yogurt imitations made from pulses, whereas coconut-based yogurt imitations presented the highest levels of SFA and salt. Among cheese imitations, differences were present in energy, protein, total fat, SFA, carbohydrates, sugars, and salt content. Vegetable oil-based cheese imitations were the lowest in protein and the highest in SFA and salt.

### 3.4. Nutrient Profile of Meat and Dairy Imitation Products

**Figure 2:**
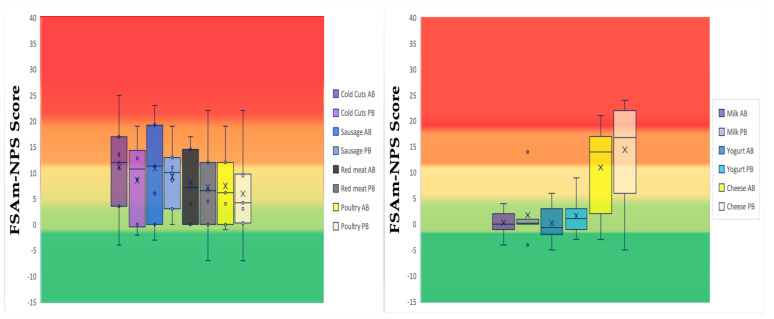
Distribution of plant-based imitations and their animal-based equivalents within the Nutri-Score categories. (Overall Boxplots of animal-based (AB) and plant-based (PB) products within the food categories analyzed. Dark green: Nutri-Score Category “A”, light green: Nutri-Score Category “B”, yellow: Nutri-Score Category “C”, light orange: Nutri-Score Category “D”, and dark orange: Nutri-Score Category “E”).

The Nutri-Score system was used to evaluate the nutrient profile of the meat and dairy imitations and to compare them with their animal-based counterparts (Figure 2). FSAm-NPS score and Nutri-Score categories A–E were estimated. No one of the products studied gained positive points for FV%.

In plant-based meat imitation products, 12% was graded as A, 16.8% as B, 30.5% as C, 35.8% as D, and 5.3% as E (Figure 2). Both plant- and animal-based meat products were most commonly graded as D, except for plant-based poultry imitations that were most commonly graded as C (Figure 2). The highest FSAm-NPS score was noticed in animal-based sausages (FSAm-NPS score= +23), followed by red meat imitations (FSAm-NPS score= +22), while the lowest were in red meat and poultry imitations (FSAm-NPS score= −7, for both cases). No differences between the means of FSAm-NPS score of plant- and animal-based meat products were detected for any of the meat categories. The prevalence of plant-based meat imitations graded as A was lower than 20%. Specifically, for poultry imitations, the prevalence of Nutri-Score Category A was 18.8%, followed by plant-based red meat imitations (16.7%) and animal-based poultry meat (10.5%). Plant-based sausage imitations and animal-based red meat did not include products classified in the Nutri-Score Category A (data not shown).

Both plant- and animal-based milk products were most commonly graded as B (Fig 2). The prevalence of products classified in the Nutri-Score Category A was higher for animal-based products (35.8%) than for plant-based milk imitations (21.3%). For yogurts, plant-based yogurt imitations were most commonly classified in Nutri-Score Category B, while animal-based were in Nutri-Score Category C. No yogurt, neither plant- nor animal-based, was classified in D or E Nutri-Score Categories. Plant-based yogurt imitations presented a higher mean of FSAm-NPS score compared to their animal-based equivalents. Plant-based cheese imitations were most commonly classified in Nutri-Score Category E, while animal-based were in Nutri-Score Category D. Seventy-one percent of plant-based cheese imitations were classified in the Nutri-Score Category E, followed by C (9.6%), D and A (7.2% for both cases), and B (4.7%). Plant-based cheese imitations also presented a higher mean of FSAm-NPS score compared to their animal-based counterparts.

## 4. Discussion

Meat and dairy imitation products are populating the Greek market in line with international trends for vegan products [14,32,33]. For all traditional meat and dairy categories, there was at least one non-animal product at supermarket shelves. All meat and dairy imitations available at supermarkets were plant-based. However, the main ingredient used differed per category and sub-category.

Cold cuts and sausage imitations were mostly wheat-based; poultry imitations were mostly soy-based, while, for red meat imitations, the use of soy, wheat, and pulses were equally common. In the case of dairy imitations, cheese was the most interesting sub-category, as it was the only category with vegetable oils as the main ingredient for 80.9% of all products. Nuts and grains were the most common ingredients for milk imitations, while, for yogurt imitations, it was nuts and soy.

The choice of ingredients impacted the protein content of products. For meat imitations, differences were seen only in sausages, in favor of wheat-based formulations. On the other hand, pulse-based formulations had the highest protein content in dairy imitations. In particular, vegetable oil-based cheeses did not contain any protein. It is worth highlighting that in our study, especially for meat imitations, the nutritional composition was similar across the same manufacturer’s brand, which may indicate that each manufacturer has a single recipe to produce multiple products (data not shown). In addition, the alternative protein and fat sources used for meat and dairy imitations sold in Greece were, almost in all cases, the same as the ones found from other studies [3,14,15,33,34].

An important finding was the nutritional composition of meat and dairy imitations, particularly in comparison with animal-based counterparts. Meat imitations did not differ in their energy content from their animal-based counterparts. Sausage imitations were lower in SFA and salt than their animal-based equivalents, yet their content in salt is still considered high. In addition, sausage imitations were higher in protein than animal-based sausages. Poultry imitations were higher in carbohydrates, and cold cuts imitations were higher in sugars than their animal-based counterparts. It is important to highlight that in meat imitation products, starch sources are used due to their properties related to texture improvement, shelf-life extension, cohesiveness, and elasticity. These properties are mostly associated with the starches’ capacity to form stable gels through gelatinization [3].

In dairy imitations, only milk imitations had a lower energy content in comparison with milk. Yogurt and cheese imitations were higher in carbohydrates and fiber, and yogurt imitations were also higher in sugars. On the contrary, milk imitations had a lower sugar content than their animal-based equivalents. Changes in carbohydrate content could be linked with the natural carbohydrate-rich nature of plant-based foods [14,15,35]; as such, the use of plant-based matrices is also linked to higher fiber content [3]. All plant-based dairy imitations categories were poorer in protein than their animal-based counterparts. The present study is in line with other countries’ studies analyzing the nutritional composition of plant-based products. For instance, the Italian non-dairy milk FLIP study [20] agrees that plant-based beverages were lower in energy and sugar compared to their animal-based counterparts. Our results also concurred with previous work that indicated a significantly lower protein content in plant-based milk imitations compared to animal-based milk products [22]. Among cheese imitations, our results agreed with others on a high SFA content [21]. However, chemical composition and nutritional value of either meat or milk from animal origin are strongly affected by animal species and animal nutrition [36,37,38].

When the nutritional quality was assessed by the Nutri-Score algorithm, in our study, the majority of plant-based beverages was classified in Nutri-Score Category “B”, in contrast with the USDA’s study that the majority was classified in “C” [21]. The classification of “meat” products in Nutri-Score Categories differed only for plant-based poultry meat alternatives that were classified as “C”, while poultry meat products were classified as “D”. In contrast with the results of Pointke M. and Pawelzik E. [39] that indicated a lower FSAm-NPS score for plant-based meat alternatives when compared with their animal-based equivalents in nine out of the thirteen meat categories (the other four did not differ significantly), our results did not indicate any differences in the FSAm-NPS score for any of the four meat categories studied.

Another important finding refers to the on-pack communication of meat and dairy imitations. In line with an Australian study, where 81% of meat imitation products are sold as vegan/vegetarian [14], in Greece, 82% of meat imitations and 60% of dairy imitations are sold as vegan/vegetarian. Nevertheless, in Greece, the presence of meat-free and dairy-free claims is also very common (60% in meat imitations and 39% in dairy imitations, respectively). Protein, fiber, vitamin, and mineral content are the main claims used in these products. However, compared to other markets, protein and micronutrient claims are less common in the Greek market [14]. Claims on fiber and allergen content (soy and gluten) are equally present in Greece as in other markets [14]. Although this study did not focus on consumer preferences and attitudes, the differences in the on-pack communication seen in Greece could be indicative of differences in target consumers, beliefs, attitudes, and knowledge.

This study also had some limitations worth highlighting. As we were using only mainstream supermarkets, we were able to identify only terrestrial plant-based meat and dairy imitations. Other novel protein sources, e.g., aquatic, insects, lab-grown, were not identified, which could highlight a market trend or require a more targeted population sampling methodology. Another limitation is linked to the standardized nutrition and ingredient declaration made on-pack, which does not always include other nutritional components, such as fiber, vitamins, minerals, percentages of fruits, pulses, vegetables, nuts, and oils. However, the lack of data is most likely linked to underestimation and does not pose great methodological risks and biases [30].

As the development and progression toward novel meat and dairy alternatives rely on consumer perceptions and acceptance, there is a need to identify and understand the stimulator factors for purchasing and consuming meat and dairy imitations. There is a need to understand whether plant-based imitations are used as a steppingstone to plant-based diets or whether the adoption of this trend in plant-based diets is here to stay and replace the traditional models. Another area of future research includes the role of meat and dairy imitations in traditional diets and in food-based dietary guidelines. Although current evidence points toward gains for health and the environment following the adoption of plant-based diets, this is based on dietary patterns that do not include such novel products. The role of those products in modern diets should be studied both in terms of protein bioavailability, but also under the scope of ultra-processed foods and their impact in health.

## 5. Conclusions

This study described meat and dairy imitation products in terms of their content, nutrition claims, quality indicators, and nutrition composition collectively and according to their main alternative protein source. Plant-based imitations have variable composition based on their main ingredient and frequently employ nutrition claims as part of their on-pack communication. Wheat and soy-based formulations can be considered suitable protein sources, while vegetable oil-based formulations do not provide any protein. When compared to their animal-based counterparts, the substitution of specific food groups with plant-based alternatives may not support an equivalent or improved diet.

## Figures and Tables

**Table 1:** Presentation of foods included in each meat and dairy imitation category eligible for the 2022 HelTH expansion.

Imitation Categories	Description
Cold cuts	Meat-free products appearing to mimic cold cuts, including “salami”, “turkey”, “ham”, “bacon”, “chorizo”, “kebab cold cut”, “carpaccio”.
Sausages	Meat-free products appearing to mimic sausages, including deep frozen sausages and cold cut sausages. Features either “sausage”, or “hot dog”, in the product name
Red meat	Meat-free products appearing to mimic red meat products, including “burgers”, “meatballs”, “mince”, “kebab”, “steak”, “souvlaki”, “gyros”.
Poultry meat	Meat-free products appearing to mimic red poultry products, including “nuggets”, “schnitzel”, “Gordon blue”, “chicken burger”, “chicken chunks”.
Milk	Dairy-free products appearing to mimic milk, including soy, almond, coconut, etc., beverages, either flavored or unflavored.
Yogurt	Dairy-free products appearing to mimic yogurt, including “yogurt”, “yogurt dessert”.
Cheese	Dairy-free products appearing to mimic cheese, including “white cheese”, “spread cheese”, “yellow cheese slices”, “yellow cheese grated”, “yellow cheese block”.

**Table 5:** Nutritional composition of meat imitation products according to the main ingredient used as an alternative source of protein.

Nutrients	Cold Cuts Imitations (*n* = 18)				Sausage Imitations (*n* = 19)				Red Meat Imitations (*n* = 43)				Poultry Meat Imitations (*n* = 16)			
	Soy-Based (*n* = 1)	Wheat-Based (*n* = 9)	Other (*n* = 8)	*p*-Value	Soy-Based (*n* = 3)	Wheat-Based (*n* = 12)	Other (*n* = 4)	*p*-Value	Soy-Based (*n* = 9)	Wheat-Based (*n* = 12)	Other (*n* = 22)	*p*-Value	Soy-Based (*n* = 8)	Wheat-Based (*n* = 3)	Other (*n* = 5)	*p*-Value
Energy (kcal)	166.0 (166.0, 166.0)	244.0 (226.5, 269.5)	197.5 (172.5, 221.8)	0.017	269.0 (237.0, -)	251.5 (213.8, 269.0)	162.5 (54.5, 223.3)	0.034	221.0 (135.0, 278.0)	222.0 (165.8, 242.2)	236.5 (202.0, 250.8)	0.820	195.5 (186.5, 221.8)	229 (212, -)	249.0 (215.5, 258.5)	0.142
Protein (g)	15.5 (15.5, 15.5)	29.3 (27.3, 33.5)	5.1 (3.5, 31.0)	0.044	14.4 (14.0, -)	29.2 (24.7, 31.1)	17.0 (7.0, -)	0.005	14.9 (14.5, 24.0)	25.5 (19.7, 27.6)	16.0 (14.5, 19.3)	0.02	11.4 (9.5, 13.8)	13.7 (10.5, -)	13.0 (6.5, 17.0)	0.832
Fat (g)	10.0 (10.0, 10.0)	12.2 (8.4, 13.5)	16.0 (6.8, 18.0)	0.192	18.0 (17.7, -)	11.8 (10.4, 13.3)	9.0 (6.8, -)	0.024	10.7 (2.8, 18.2)	9.9 (3.2, 12.4)	14.3 (11.5, 16.6)	0.020	7.9 (6.5, 10.8)	7.8 (7.6, -)	15.3 (4.2, 17.5)	0.689
SFA (g)	2.6 (2.6, 2.6)	2.6 (1.1, 5.9)	1.5 (1.3, 2.1)	0.599	3.5 (2.2, -)	1.2 (0.9, 1.3)	7.0 (0.9, -)	0.186	4.0 (0.4, 9.3)	1.2 (0.8, 1.8)	5.3 (1.1, 9.5)	0.090	1.2 (0.7, 3.1)	0.8 (0.8, -)	1.1 (0.6, 12.6)	0.812
Carbohydrates (g)	3.0 (3.0, 3.0)	5.4 (3.0, 6.5)	5.9 (2.0, 7.0)	0.737	4.0 (3.0, -)	4.5 (3.6, 5.8)	4.3 (3.0, -)	0.749	5.0 (2.9, 14.8)	6.6 (2.8, 12.0)	7.7 (4.7, 10.4)	0.785	16.6 (12.8, 20.0)	20.8 (15.7, -)	14 (11.6, 15.3)	0.091
Sugars (g)	2.0 (2.0, 2.0)	1.5 (0.4, 2.4)	1.9 (0.9, 2.8)	0.763	1.1 (0.7, -)	0.6 (0.4, 2.0)	0.5 (0.0, -)	0.097	0.9 (0.5, 1.1)	1.5 (1.1, 2.7)	0.8 (0.6, 2.5)	0.293	0.9 (0.5, 4.6)	2.7 (0.5, -)	1.6 (1.4, 2.1)	0.450
Fiber (g)	-	-	4.4 (4.1, 5.0)	-	2.9 (0.0, -)	(2.4, -)	3.2 (3.2, 3.2)	1.000	5.7 (2.6, 12.5)	4 (1.5, -)	4.1 (2.0, 5.3)	0.649	2.8 (1.6, 6.5)	5.0 (5.0, 5.0)	4.7 (3.2, -)	0.759
Salt (g)	1.9 (1.9, 1.9)	1.9 (1.4, 2.5)	2.4 (2.1, 2.8)	0.334	1.9 (1.4, -)	1.6 (1.4, 1.8)	1.3 (1.2, -)	0.054	1.3 (0.9, 1.4)	1.7 (1.3, 1.9)	1.3 (1.0, 1.7)	0.096	1.3 (1.1, 1.5)	0.9 (0.9, -)	1.4 (1.1, 1.9)	0.638

Values shown are median (Q1, Q3). Bold remarks the specific categories with a statistic significant difference.

**Table 6:** Nutritional composition of dairy imitation products according to the main ingredient used as an alternative source of protein or fat.

Nutrients	Milk Imitations (*n* = 221)						Yogurt Imitations (*n* = 40)					Cheese Imitations (*n* = 80)				
	Nut-Based (*n* = 64)	Coconut-Based (*n* = 18)	Grain-Based (*n* = 62)	Pulse-Based (*n* = 20)	Mixed (*n* = 16)	*p*-Value	Nut-Based (*n* = 14)	Coconut-Based (*n* = 8)	Grain-Based (*n* = 6)	Pulse-Based (*n* = 12)	*p*-Value	Nut-Based (*n* = 2)	Pulse-Based (*n* = 9)	Mixed (*n* = 3)	Vegetable oil Based (*n* = 66)	*p*-Value
Energy (kcal)	37.0 (27.0, 51.0)	30.5 (20.0, 40.3)	55.0 (47.8, 61.0)	50.0 (41.0, 61.0)	42.0 (28.8, 56.8)	<0.001	79.5 (57.8, 110.0)	91.0 (76.8, 111.3)	94.0 (84.8, 96.5)	75.0 (59.8, 80.8)	0.154	438.0 (429.1, -)	165.0 (120.0, 200.5)	181.0 (166.0, -)	285.0 (277.0, 305.0)	<0.001
Protein (g)	0.8 (0.6, 1.0)	0.2 (0.1, 0.4)	0.7 (0.5, 1.0)	3.3 (3.0, 3.7)	0.5 (0.4, 1.0)	<0.001	1.9 (0.8, 2.3)	0.6 (0.6, 2.1)	1.7 (1.2, 2.1)	3.8 (3.6, 3.9)	0.001	7.9 (7.1, -)	16.0 (13.0, 18.5)	1.7 (2.5, -)	0.0 (0.0, 0.5)	<0.001
Fat (g)	1.9 (1.4, 2.5)	1.7 (1.2, 2.7)	1.3 (1.0, 1.5)	2.2 (1.8, 2.5)	1.6 (0.9, 2.3)	<0.001	3.4 (1.7, 5.2)	4.4 (3.0, 5.3)	2.1 (0.9, 3.2)	2.2 (2.1, 2.3)	0.032	40.2 (39.4, -)	9.0 (7.0, 13.0)	16.3 (15.3, -)	23.0 (21.0, 24.0)	<0.001
SFA (g)	0.2 (0.1, 0.3)	1.4 (1.0, 2.5)	0.2 (0.2, 0.3)	0.4 (0.3, 0.5)	0.2 (0.1, 0.9)	<0.001	0.4 (0.1, 0.5)	3.8 (1.7, 4.5)	0.3 (0.1, 0.4)	0.3 (0.3, 0.4)	0.001	16.0 (10.1, -)	1.6 (1.2, 2.2)	1.4 (1.2, -)	21.0 (18.4, 21.0)	<0.001
Carbo-hydrates (g)	3.1 (1.5, 6.9)	2.0 (1.6, 3.6)	9.6 (7.7, 11.0)	3.2 (1.0, 6.4)	4.0 (1.5, 9.5)	<0.001	11.2 (4.7, 15.9)	10.1 (6.1, 15.4)	15.0 (13.4, 20.0)	9.9 (2.9, 12.0)	0.049	40.9 (4.9, -)	1.1 (0.8, 2.0)	9.7 (0.3, -)	21.9 (15.7, 23.0)	<0.001
Sugars (g)	2.4 (0.3, 4.1)	1.5 (0.5, 2.6)	5.6 (4.4, 7.5)	3.3 (1.6, 6.3)	1.4 (0.2, 4.6)	<0.001	1.9 (0.6, 11.0)	8.1 (1.5, 12.1)	8.5 (4.4, 11.7)	9.2 (1.4, 11.0)	0.825	1.6 (0.1, -)	0.5 (0.5, 0.6)	0.3 (0.3, -)	0.0 (0.0, 0.1)	<0.001
Fiber (g)	0.8 (0.4, 1.6)	0.2 (0.1, 0.6)	0.8 (0.5, 1.2)	0.6 (0.5, 0.9)	0.2 (0.1, 0.5)	0.001	1.1 (0.8, 1.6)	0.8 (0.3, -)	1.0 (0.5, 1.1)	0.5 (0.5, 0.5)	0.421	-	2.0 (2.0, 2.0)	2.1 (0.4, -)	1.7 (0.5, -)	0,953
Salt (g)	0.1 (0.1, 0.1)	0.1 (0.1, 0.1)	0.1 (0.1, 0.1)	0.1 (0.1, 0.2)	0.1 (0.1, 0.1)	0.053	0.1 (0.0, 0.1)	0.2 (0.2, 0.4)	0.1 (0.1, 0.1)	0.1 (0.1, 0.1)	0.010	1.1 (1.0, -)	1.4 (0.1, 1.8)	1.0 (0.8, -)	2.0 (1.8, 2.2)	<0.001

Values shown are median (Q1, Q3). Bold remarks the specific categories with a statistic significant difference.

## Data Availability

HelTH is available at https://www.eurofir.org/our-tools/foodexplorer/ (accessed on 23 December 2022).
